# Epidemiological characteristics and behaviors of online broadcast suicidality in China: implications for targeted prevention strategies

**DOI:** 10.3389/fpubh.2024.1396460

**Published:** 2024-05-07

**Authors:** Chun-ya Li, Yu Xiao, Ting-ting Chen, Shao-yi Zhu

**Affiliations:** ^1^Psychosomatic Medical Center, The Fourth People's Hospital of Chengdu, Chengdu, China; ^2^Psychosomatic Medical Center, The Clinical Hospital of Chengdu Brain Science Institute, MOE Key Lab for Neuroinformation, University of Electronic Science and Technology of China, Chengdu, China; ^3^Mental Health Center, West China Hospital of Sichuan University, Chengdu, China; ^4^Department of Psychiatry, Shantou University Mental Health Center, Shantou, China

**Keywords:** China, internet users, suicide, suicide attempt, suicide broadcast

## Abstract

**Objectives:**

Suicide is a global health concern, exacerbated by stigma around mental illnesses. Online platforms like Twitter and Sina Weibo have seen a rise in “online broadcast suicide,” where individuals share suicidal thoughts and actions. However, there is limited understanding of the epidemiological characteristics, particularly in China. This study aims to analyze the demographics and behaviors of individuals engaging in online broadcast suicide in China to inform targeted prevention strategies.

**Methods:**

A total of 525 incidents were identified through systematic retrieval of relevant news reports from online sources. Subsequently, a content analysis was performed on these reports to extract detailed information on the characteristics of each individual incident.

**Results:**

Among the incidents analyzed, the male-to-female ratio was 1:1.6, with an average age of 23.1 ± 5.9 years. Approximately 71.9% took place in Southern China. Unemployment was reported in 15.0% of incidents. Relationship breakup (62.3%) was cited as the leading cause of suicide. Wrist cutting (58.2%) emerged as the predominant suicide method, and home (36.2%) was the most common location for these tragic events. Instant messaging apps were the primary platforms (54.7%) for conveying suicidal thoughts and actions. Additionally, among the 525 incidents examined, 12.0% disclosed having a mental disorder, and 7.6% had a history of prior suicide attempts. Significant variations were observed across age, gender, region, and occupation categories.

**Conclusion:**

This study emphasizes the importance of developing suicide prevention programs for internet users. Besides, interventions should be customized to meet the specific needs of various populations.

## 1 Introduction

According to recent estimates by the World Health Organization (WHO), suicide continues to be a major global cause of mortality (1). Timely intervention has the potential to significantly reduce suicide-related deaths (2). Unfortunately, mental illnesses, including suicidal behaviors, often carry a stigma that undermines their seriousness and is sometimes dismissed as mere attention-seeking (3). This stigma further hampers individuals from seeking proper treatment, resulting in an elevated risk of suicide and reduced chances of receiving timely assistance (4).

In the present day, the Internet has become an integral component of many people’s everyday lives. Online platforms such as Twitter and Sina Weibo have provided users with interactive spaces where they feel more comfortable disclosing personal information (5). Consequently, individuals with suicidal tendencies may be motivated to share and communicate their thoughts and behaviors related to suicide online (6). This includes cases of livestreamed suicides, wherein individuals use various media formats (e.g., pictures, text messages, and videos) to broadcast their intentions and actions before taking their own lives, often referred to as “online broadcast suicidality” (5). The term “suicidality” encompasses all thoughts and actions related to suicide, including ideation, attempts, and completed suicides (7). As an increasing number of individuals with suicidal tendencies engage online, there is an amplified potential for interventions, prevention efforts, and crisis responses (6). Real-time notifications can alert other internet users when someone broadcasts their suicidality. By appropriately responding and offering support in these situations, there is a higher likelihood of mitigating the risk of fatal outcomes (6).

In order to enhance public responses to online broadcasts of suicidality, past research has primarily focused on examining public knowledge and beliefs regarding such broadcasts (5, 8). Nevertheless, it is equally crucial to comprehend the attributes of individuals who employ the internet for reasons related to suicide. Despite existing studies in this domain (9, 10), there remains a lack of comprehensive research concerning the epidemiological characteristics of individuals who engage in broadcasting their suicidal tendencies online. Consequently, the aim of this study is to conduct a systematic analysis of the epidemiological characteristics associated with online broadcasts of suicidality specifically within the context of China.

## 2 Methods

**Figure 1:**
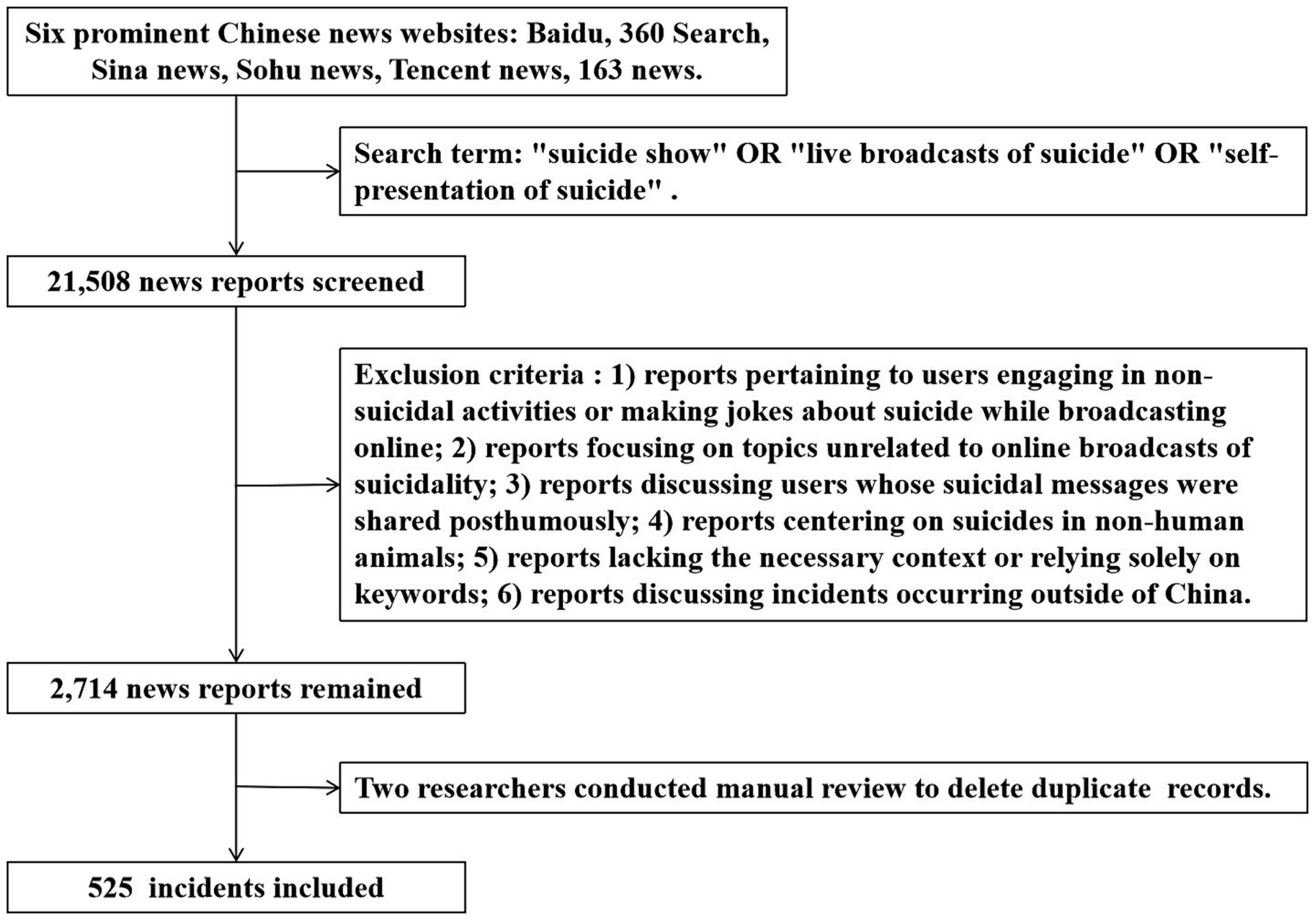
Flow chart of news report collection.

The research procedure entailed three sequential steps: (1) data acquisition, (2) data preprocessing, and (3) data analysis. Figure 1 illustrates the procedures involved in data collection and preprocessing.

### 2.1 Data collection

To establish a comprehensive dataset, an extensive collection of news reports was conducted on February 5, 2023. This involved searching six prominent Chinese news websites: Baidu,^1^ 360 Search,^2^ Sina news,^3^ Sohu news,^4^ Tencent news,^5^ and 163 news.^6^ The search criteria encompassed keywords related to the topic, including “suicide show” (“自杀秀,” “自杀展示”), “live broadcasts of suicide” (“直播自杀,” “自杀直播”), and “self-presentation of suicide” (“晒自杀,” “秀自杀”). The determination of these key search terms was based on the following two steps. First, in order to maximize the relevant information related to online broadcast suicide, we conducted exploratory searches on the aforementioned six news websites to identify a list of relevant search terms. Second, we extensively reviewed literature on the subject to document existing effective keywords. Subsequently, after comprehensive review and consideration by our research team, we finalized these search keywords. Consequently, a total of 21,508 news reports accompanied by the aforementioned keywords were acquired as a result of the search.

### 2.2 Data preprocessing

Following the completion of data collection, preprocessing procedures were implemented to prepare the raw data for subsequent analysis. To eliminate irrelevant news reports, a manual examination of the collected data was carried out. This study defined irrelevant reports as those falling under the following categories: (1) reports pertaining to users engaging in non-suicidal activities or making jokes about suicide while broadcasting online; (2) reports focusing on topics unrelated to online broadcasts of suicidality, such as movie or real-world instances of suicidality; (3) reports discussing users whose suicidal messages were shared posthumously; (4) reports centering on suicides in non-human animals, like cats; (5) reports lacking the necessary context or relying solely on keywords, which hindered accurate coding; and (6) reports discussing incidents occurring outside of China. The assessment of all 21,508 news reports was conducted by two independent coders. Any report that both coders agreed to exclude was subsequently eliminated from consideration. In cases where discrepancies arose between the two coders, a third researcher was consulted to make a final decision. Following the removal of 18,794 irrelevant reports, a total of 2,714 reports remained for further analysis. To eliminate duplicate records, integration procedures were implemented, resulting in the removal of 2,189 redundant news reports. Ultimately, a collection of relevant news reports comprising 525 incidents was obtained.

### 2.3 Data analysis

Following data preprocessing, an in-depth content analysis was conducted by two independent coders on the relevant reports to extract information regarding the characteristics of each individual incident. The extracted information comprises two major components: demographic characteristics, including gender, age, geographical location, and employment status, as well as detailed aspects of the incidents, such as the time of occurrence, location of suicide, method used, reasons behind the act, internet platforms broadcasting suicidality, mental health status, history of suicide, and level of suicidality. The extent of interrater agreement between the coders was assessed using Cohen’s kappa coefficient, with values equal to or exceeding 0.6 considered indicative of acceptable reliability. The final Kappa scores ranged from 0.8 to 1.0, reflecting substantial to perfect agreement. Any discrepancies that arose during the analysis were resolved through rigorous team discussions to attain consensus.

For data analysis in this study, we employed the IBM SPSS Statistics 22.0 and XLSTAT 2020 software. To explore the dataset’s characteristics, descriptive analyses and Mann-Kendall trend tests were performed. A battery of statistical tests was conducted to examine age, gender, regional, and occupational differences across the various categories. These tests included chi-squared tests for categorical variables, independent-sample *t*-tests for continuous variables, and 1-way ANOVA (followed by post-hoc Scheffe’s tests) for multiple group comparisons. All statistical analyses were performed using two-tailed tests, and a significance level of 0.05 was set. Given that a few incidents may have multiple values within a single category, independent sample comparisons are not feasible. Thus, these incidents were excluded from the corresponding analysis for each category.

## 3 Results

### 3.1 Basic demographic characteristics

The final analysis comprised a total of 525 incidents, each involving a distinct individual. Table 1 presents the demographic characteristics of the sample. Of the 525 incidents, 516 reported their gender, resulting in a male to female ratio of 1:1.6. Age information was available for 308 incidents, with an average age of 23.1 ± 5.9 years (females, 22.4 ± 5.0 years; males, 24.3 ± 6.8 years). The age range varied from 15 to 53 years, with no significant gender differences observed. Regarding location, 501 incidents provided information on their occurrence. Among these, 44.1% (*n* = 221) were located in Eastern China, while 71.9% (*n* = 360) were concentrated in Southern China when considering the broader geographical regions. No significant age or gender disparities were found. Furthermore, 226 individuals disclosed their employment status. Among them, 34 incidents (15.0%) involved unemployed individuals. No significant age, gender, or regional distinctions were evident in this regard.

**Table 1:** Demographic information (*n* = 525).

Category	*n*	%
**Gender (*n* = 516)**		
Female	314	60.9
Male	202	39.1
Age (years, *n* = 308)		
15 ~ 19	50	16.2
20 ~ 24	135	43.8
25 ~ 29	68	22.1
30 ~ 34	36	11.7
35 ~ 39	14	4.6
≥ 40	5	1.6
**Employment status (*n* = 226)**		
Employed	162	71.7
Unemployed	34	15.0
Student	30	13.3
**Province (*n* = 501)**		
Jiangsu	55	11.0
Guangdong	53	10.6
Sichuan	47	9.4
Shandong	47	9.4
Fujian	40	8.0
Zhejiang	34	6.8
Hubei	32	6.3
Anhui	26	5.1
Shaanxi	21	4.2
Henan	16	3.2
**Province (*n* = 501)**		
Hebei	16	3.2
Shanghai	16	3.2
Beijing	15	3.0
Chongqing	14	2.8
Hunan	12	2.4
Hong Kong	7	1.4
Guangxi	7	1.4
Liaoning	7	1.4
Taiwan	5	1.0
Guizhou	5	1.0
Tianjin	4	0.8
Jiangxi	3	0.6
Shanxi	3	0.6
Heilongjiang	3	0.6
Jilin	2	0.4
Yunnan	2	0.4
Gansu	2	0.4
Hainan	2	0.4
Ningxia	1	0.2
Qinghai	1	0.2
Tibet	1	0.2
Xinjiang	1	0.2
Inner Mongolia	1	0.2

### 3.2 Time of occurrence

**Figure 2:**
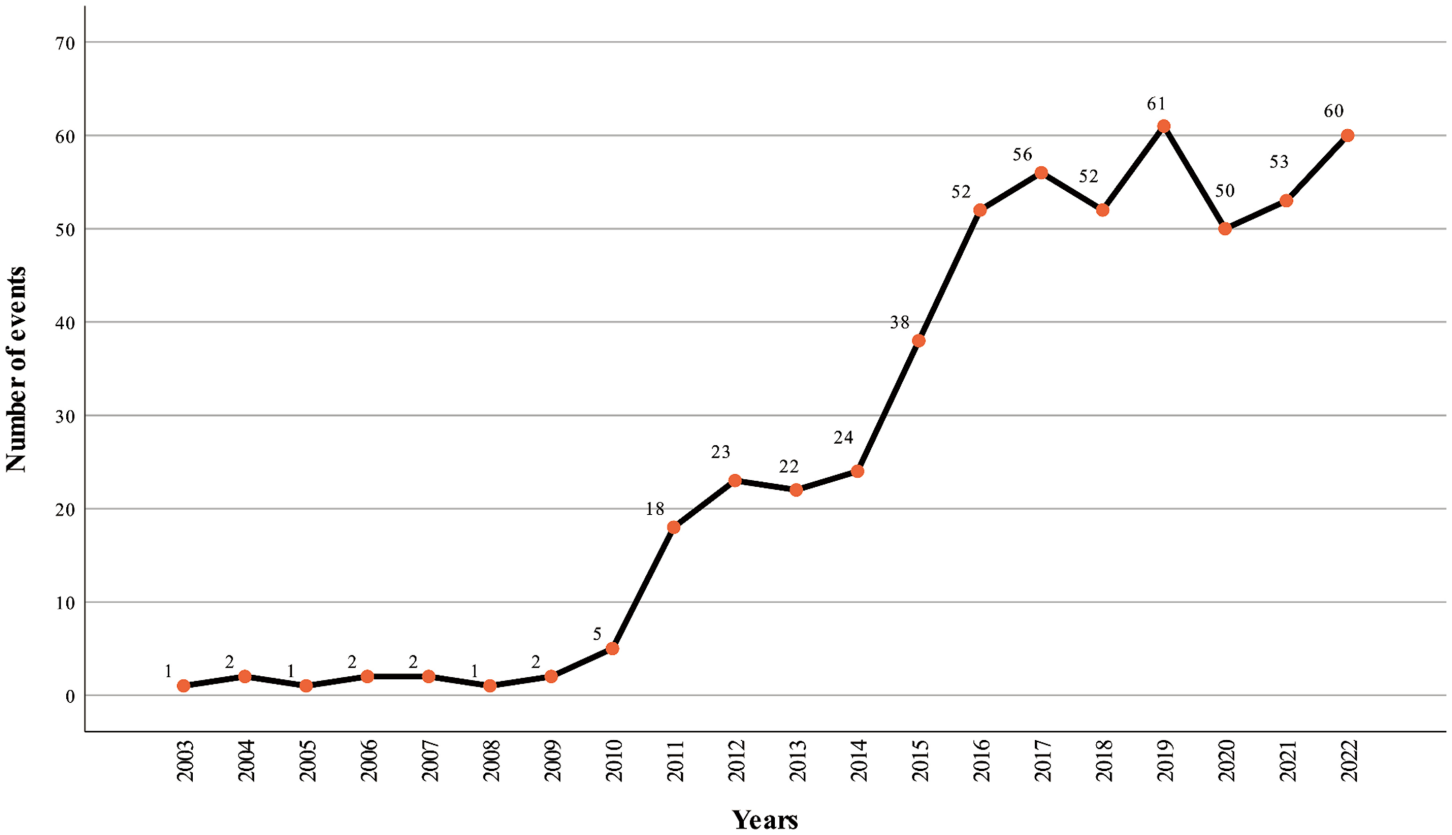
The number of incidents between 2003 and 2022.

Based on the Mann-Kendall trend test (*S* = 154, Z = 4.99, *p* < 0.001), a significant increase in the frequency of incidents was observed from 2003 to 2022 (Figure 2). This increasing trend was consistent across gender, age, region, and occupation groups, with the exception of residents from Northeast China (*p* > 0.05), and students (*p* > 0.05). Regarding the monthly distribution, May had the highest frequency of incidents, accounting for 67 cases (12.8%). No significant variations were found when comparing different genders, age groups, regions, and occupations. Analyzing the hourly patterns, incidents were most frequently recorded between 18:00 and 00:00, with 207 cases (39.4%) occurring during this period. Again, no noteworthy disparities emerged across gender, age, region, or occupation.

### 3.3 Suicide locations

In this study, 345 incidents were found to have identifiable suicide locations (refer to Table 2). Among these incidents, the overwhelming majority (*n* = 340; 98.6%) were associated with a single location, while only 5 incidents (1.4%) were linked to two distinct locations. The most frequently reported suicide location was at home, which accounted for 36.2% (*n* = 125) of the total cases. In our analysis, significant gender differences in suicide locations were observed (Fisher exact test: *p* < 0.01). Specifically, females exhibited a higher likelihood of choosing rental places as the site for suicide compared to males (*χ*^2^ = 7.6, *p* < 0.01). There are no significant differences in age, region or occupation.

**Table 2:** Suicide methods, reasons, locations, and types of internet platforms used for broadcasting suicidality.

Category	*n*	%
**Suicide methods (*n* = 490)^a^**		
Slitting wrists	285	58.2
Taking medications or poisons	164	33.5
Jumping from a height	43	8.8
Charcoal burning	23	4.7
Drowning	12	2.4
Exposure to gas	10	2.0
Hanging	9	1.8
Exposure to poisonous animals	4	0.8
Motor vehicle collision	3	0.6
Setting fire	2	0.4
**Reasons for suicide (*n* = 478)^b^**		
Relationship breakup	298	62.3
Family conflicts	100	20.9
Job-related stress	55	11.5
Mental disorder	48	10.0
Financial difficulties	38	7.9
Physical illness	13	2.7
Encountering discrimination and bullying	11	2.3
Academic pressure	10	2.1
loss of close relationships	4	0.8
**Type of platform (*n* = 497)^c^**		
Instant messaging apps	272	54.7
Social networking websites	159	32.0
Online bulletin boards	86	17.3
Online chat rooms	7	1.4
Video-sharing websites	3	0.6
**Suicide locations (*n* = 345)^d^**		
Home^e^	125	36.2
Rental place	84	24.3
Hotel	66	19.1
Dormitory	13	3.8
Internet café	12	3.5
Others	51	14.8

^a^Occurrences involving multiple suicide methods were tabulated separately within their respective subcategories. ^b^Occurrences involving multiple suicide causes were tabulated separately within their respective subcategories. ^c^Occurrences involving multiple types of internet platforms were tabulated separately within their respective subcategories. ^d^Occurrences involving multiple suicide locations were tabulated separately within their respective subcategories. ^e^Home denotes an individual’s permanent place of residence.

### 3.4 Suicide methods

Among the 525 incidents analyzed, suicide methods were identifiable in 490 cases (Table 2). Out of these, 433 incidents (88.4%) were associated with a single method, while 57 incidents (13.2%) involved two different methods. The most frequently employed suicide method was wrist cutting, accounting for 58.2% (*n* = 285) of the total cases. Our analysis revealed significant age (*F* = 3.9, *p* < 0.01) differences in suicide methods. More specifically, individuals who committed suicide by hanging tended to be older compared to those who used medications or poisons (*p* < 0.01, post-hoc Scheffe’s test), slit their wrists (*p* < 0.01, post-hoc Scheffe’s test), or jumped from a high place (*p* < 0.05, post-hoc Scheffe’s test). There are no significant differences in gender, region and occupation.

### 3.5 Reasons for suicide

In our study, suicide causes were identified in 478 cases (Table 2). Among these cases, 386 incidents (80.8%) were linked to a single cause, while 69 incidents (14.4%) and 23 incidents (4.8%) involved two and three distinct causes, respectively. The primary cause reported was relationship breakup, accounting for 62.3% (*n* = 298) of the total cases. Our analysis revealed significant gender differences in suicide causes (*p* < 0.001, Fisher exact test). More specifically, males were found to have a higher likelihood of committing suicide as a result of financial difficulties compared to females (*p* < 0.001, Fisher exact test). There are no significant differences in age, region or occupation.

### 3.6 Internet platforms

Out of the 525 incidents examined, 497 cases (94.7%) involved specific types of internet platforms through which suicidality was broadcasted (Table 2). Among these cases, 482 incidents (97.0%) were associated with the use of a single platform type, while 15 incidents (3.0%) involved the use of two different types of platforms. The most commonly utilized platform for broadcasting suicidality was instant messaging apps, accounting for 54.7% (*n* = 272) of the total cases.

Our analysis uncovered significant gender (*p* < 0.001, Fisher exact test), regional (*p* < 0.01, Fisher exact test), and occupational (*p* < 0.01, Fisher exact test) disparities in the usage of internet platforms for broadcasting suicidality. More specifically, males were more likely than females to employ online bulletin boards for this purpose (*χ*^2^ = 12.5, *p* < 0.001). Individuals from Southern China demonstrated a greater preference for instant messaging apps compared to those from Northern China (*χ*^2^ = 4.8, *p* < 0.05). Moreover, employed individuals were more inclined to using instant messaging apps, as opposed to students (*χ*^2^ = 5.1, *p* < 0.05). No significant variations were found when comparing different ages.

### 3.7 Mental health status and suicidal history

In this study, 63 cases documented the presence of mental disorders. Out of these cases, 92.1% (*n* = 58) were associated with a single type of mental disorder, whereas only 5 incidents (7.9%) involved two distinct types of mental disorders. Specifically, 57 individuals (90.5%) reported suffering from depressive disorder, 5 individuals (7.9%) reported anxiety disorders, and 3 individuals (4.8%) reported personality disorders. There were 3 cases where a specific type of mental disorder was not reported. There are no significant differences in age, gender, region or occupation. Furthermore, 7.6% of the incidents (40/525) disclosed a history of previous suicide attempts. No significant disparities emerged across age, gender, region, or occupation.

### 3.8 Levels of suicidality

In this study, 491 incidents revealed varying degrees of suicidal tendencies. Among them, 420 incidents (85.5%) were classified as suicide attempts, followed by suicidal ideation (*n* = 39; 8.0%) and suicide (*n* = 32; 6.5%). Significant disparities in levels of suicidality were observed across different occupations (*p* < 0.05, Fisher exact test). Employed individuals were found to have a higher likelihood of being associated with suicide attempts compared to those who were students (*p* < 0.05, Fisher exact test) and unemployed (*p* < 0.05, Fisher exact test). There are no significant differences in age, gender, or region.

## 4 Discussion

Lyu et al. (11) used official suicide mortality data provided by the Chinese Center for Disease Control and Prevention to examine the patterns of suicide across 33 provincial-level regions in China based on gender and age. Their results indicated that over the past 30 years, the overall suicide rate in China has gradually declined, attributed to the rapid development of society, economy, and education (11). However, fast-paced lifestyles and high levels of internal migration may exacerbate other new sources of stress (12). Of concern is that online suicide attempts and completion rates appear to be increasing, showcasing the individual’s internal struggles encompassing feelings of dissatisfaction, insecurity, isolation, and negative self-perception (5, 13, 14). Our study systematically examined the epidemiological characteristics of online broadcasts of suicidality in China, providing valuable evidence and insights for the development of future public awareness campaigns. By analyzing accounts of these incidents, our findings shed light on a deeper understanding of individuals with suicidal tendencies and, perhaps more importantly, how suicide can be prevented within an online environment.

To begin with, it is noteworthy to mention the demographic characteristics associated with online broadcasts of suicidality. In our study, a higher number of incidents were observed among females, aligning with previous findings suggesting an increased risk of suicide among females in China (15, 16). However, in offline suicide studies, the male-to-female ratio of suicide rates has been steadily increasing, reaching 1.56 in 2017 (11). The decrease in female suicide rates offline is related to the establishment of social networks, improvement in social and family status for women (11). One possible explanation for the different trends in online and offline suicide is the presence of gender-specific coping mechanisms and social support structures that differ between online and offline environments. Moreover, the majority of incidents (82.1%) occurred within the age group of 15 to 29 years, reflecting a persistently high prevalence of suicide risk within this specific age cohort of the Chinese population (17, 18). In terms of regional distribution, we found that a considerable portion of incidents took place in Eastern China (44.1%) and Southern China (71.9%). These figures are consistent with the proportions of internet users in each respective region (19). This correlation implies that the level of internet accessibility may play a role in influencing the distribution of online broadcasts of suicidality among different populations.

Second, regarding incident details, we found that a significant proportion (39.4%) of incidents occurred during the time frame of 18:00 to 00:00, which can be considered a high-risk period for suicide attempts (20). Notably, our study reports wrist cutting as the most prevalent method used, deviating from previous research findings (21, 22). Several factors could be speculated as potential explanations for this discrepancy. It is essential to consider that cultural norms and societal attitudes toward suicide can vary across regions and countries, leading to variations in the choice of methods employed (21). Variability in data collection techniques, such as reliance on self-reporting or medical records, could also contribute to divergent findings. Furthermore, public awareness campaigns, prevention efforts, or changes in access to lethal means can influence the popularity of certain suicide methods over time. Differences in the timing of data collection across studies might account for the observed inconsistencies (22). In terms of suicide location, the majority of incidents took place at home, highlighting the significance of personal surroundings in suicidal behaviors. Relationship breakup emerged as the leading cause of suicide, underscoring the impact of interpersonal relationships on individuals’ mental well-being in this context (23). Instant messaging apps were identified as the primary medium for broadcasting suicidal thoughts and actions, shedding light on the role of modern communication technologies in facilitating such behaviors (24). Only a minority (12.0%) of incidents reported a history of psychiatric disorders, which is remarkable given prior studies indicating a higher prevalence (32.3%) of psychiatric comorbidities among suicide attempters (25). This discrepancy might be attributed to inadequate awareness and understanding of mental health issues among the Chinese population, resulting in an underestimation of psychiatric disorder rates (26). Further research is warranted to grasp the complex interplay between sociocultural factors, mental health literacy, and suicidal behaviors in this particular context.

Third, in China, there is limited availability of professional mental health services and suicide prevention training (27). Online suicide prevention has shown promise in previous studies (5, 28), as compassionate individuals within the online community have demonstrated a willingness to take positive actions. However, their knowledge and understanding of effective responses may be inadequate (5). Expanding the use of social media for suicide prevention training could be a cost-effective approach, considering its effectiveness and wide reach (29). To maximize the impact, it is important to make best-practice materials more accessible in multiple languages and distribute them across various social media platforms, not solely targeting professional organizations (30). Leveraging popular platforms such as search engine sites, Weibo, and other easily accessible websites can ensure widespread dissemination of this information within the social media environment (6). Furthermore, social media platforms can serve as a valuable avenue for skilled individuals to provide online support to those in need (31, 32). It is also crucial for emergency services to establish clear protocols for gathering information and responding to life-threatening crises (5).

## 5 Limitations

This study has several limitations that need to be acknowledged. First, given the way the research was done, there are certain limitations in terms of diversity and incompleteness of the data, as news services do not always include same information, and conceal some due to ethics and space limitations. Second, social media users cannot be representative of the entire population in China, which means the results might not be applicable to nonusers. Third, online suicide incidents could potentially vary across different online platforms due to variations in user environments, communication dynamics, and other contextual factors. Finally, within the scope of this study, there were a mere 58 incidents with complete data, precluding the possibility of conducting the necessary multivariate analysis. In future research, it would be beneficial to make a correlation of the observed variables, in order to better understand the relevant internal and external factors that increase the risk of this kind of behavior.

## 6 Conclusion

This study emphasizes the importance of developing suicide prevention programs for internet users. To effectively address the heterogeneity in characteristics and demographics observed in online broadcasts of suicidality, interventions should be customized to meet the specific needs of various populations.

## Data availability statement

The original contributions presented in the study are included in the article/supplementary material, further inquiries can be directed to the corresponding author.

## Ethics statement

Ethical approval was not required for the study involving humans in accordance with the local legislation and institutional requirements. Written informed consent to participate in this study was not required from the participants or the participants’ legal guardians/next of kin in accordance with the national legislation and the institutional requirements.

## Author contributions

C-yL: Data curation, Writing – original draft. YX: Data curation, Investigation, Writing – review & editing. T-tC: Investigation, Writing – review & editing. S-yZ: Investigation, Writing – review & editing.

## Footnotes

^1^ https://www.baidu.com/

^2^ https://www.so.com/

^3^ http://news.sina.com.cn/

^4^ http://news.sohu.com/

^5^ http://news.qq.com/

^6^ http://news.163.com/
